# Emerging therapeutic approaches in graves’ ophthalmopathy: an update on pharmacological interventions

**DOI:** 10.3389/fimmu.2025.1647602

**Published:** 2025-07-25

**Authors:** Lin Wang, Linlin Chen

**Affiliations:** Department of Ophthalmology, Fourth People`s Hospital of Shenyang, Shenyang, Liaoning, China

**Keywords:** graves’ ophthalmopathy, corticosteroids, immunosuppressants, teprotumumab, drug therapy

## Abstract

Graves’ ophthalmopathy (GO), also known as thyroid eye disease (TED), is the most common extrathyroidal manifestation of Graves’ disease and a leading cause of visual morbidity. The disease primarily affects the orbital tissue and is characterized by inflammation, expansion of extraocular muscles, and remodeling of orbital fat, resulting in proptosis, diplopia, and even vision loss. Active GO poses significant therapeutic challenges and often requires prompt intervention to preserve visual function and improve quality of life. Over the past decade, considerable progress has been made in understanding the immunopathogenesis of GO, leading to the development of targeted pharmacological therapies that extend beyond traditional systemic corticosteroids. This review summarizes recent advances in the drug therapy of active GO, focusing on novel immunomodulators, biological agents such as monoclonal antibodies targeting CD20, IL-6 R, and insulin-like growth factor-1 receptor (IGF-1R), and evolving treatment strategies based on disease activity and severity. We also discuss current clinical practice guidelines, emerging therapeutic targets under investigation, and future perspectives in the individualized management of this vision-threatening autoimmune condition.

## 1 Introduction

Graves’ ophthalmopathy (GO), or thyroid-associated ophthalmopathy (TAO), is an autoimmune inflammatory disorder of the orbit that commonly coexists with Graves’ hyperthyroidism. It affects up to 20% of patients with Graves’ disease and significantly impacts functional vision, appearance, and psychosocial well-being(!!! INVALID CITATION!!! (1)). GO is marked by autoantibody-mediated activation of orbital fibroblasts, infiltration of immune cells, and overproduction of glycosaminoglycans, leading to tissue expansion, proptosis, and, in severe cases, compressive optic neuropathy (1, 2).

Based on the European Group on Graves’ Orbitopathy (EUGOGO) consensus, GO is categorized by disease activity (active vs. inactive) and severity (mild, moderate-to-severe, or sight-threatening) (3, 4). Active GO is particularly challenging due to persistent inflammation, progressive disfigurement, and the potential for irreversible visual impairment (5). Although systemic glucocorticoids have long been the mainstay of treatment, their efficacy is limited in some patients, and relapse is common after withdrawal. Additionally, high-dose steroids carry considerable systemic toxicity, prompting the search for safer and more effective therapies (6).

Recent years have witnessed a paradigm shift in GO treatment strategies, driven by deeper insights into disease pathophysiology and advances in immunotherapy. Biological agents targeting specific pathways, including anti-CD20 monoclonal antibodies (e.g., rituximab or obinutuzumab) (7), IL-6 receptor antagonists (e.g., tocilizumab) (8), and insulin-like growth factor-1 receptor (IGF-1R) inhibitors (e.g., teprotumumab) (9), have shown promising efficacy in clinical trials. Moreover, treatment algorithms are increasingly emphasizing early intervention during the active phase to prevent progression and optimize long-term outcomes.

This review provides a comprehensive update on recent pharmacologic advances in the management of active GO. It highlights the clinical efficacy, safety profiles, and mechanistic rationale of emerging therapies, discusses evolving treatment guidelines, and explores ongoing challenges and future directions in GO care.

## 2 Pathophysiology of graves’ ophthalmopathy

### 2.1 Autoantigens and immune activation

Two key autoantigens have been identified in GO pathogenesis: the thyroid-stimulating hormone receptor (TSHR) and the IGF-1R (10, 11). Orbital fibroblasts and preadipocytes express both TSHR and IGF-1R, forming a functional complex. Autoantibodies, particularly thyroid-stimulating immunoglobulins (TSIs), bind to TSHR on orbital fibroblasts, triggering proinflammatory signaling and adipogenic differentiation. Recent studies have shown that IGF-1R, when stimulated by its ligands or cross-talked with TSHR signaling, enhances fibroblast activation, hyaluronan synthesis, and proinflammatory cytokine secretion (12). This mechanism underlies the rationale for IGF-1R-targeted therapies, such as teprotumumab.

### 2.2 Orbital fibroblasts and adipogenesis

Orbital fibroblasts are the primary effector cells in GO. Upon activation by TSIs, IGF-1, or proinflammatory cytokines (e.g., IL-1β, TNF-α), these cells proliferate, secrete glycosaminoglycans like hyaluronic acid, and differentiate into either myofibroblasts or adipocytes, depending on their CD90 expression. CD90 (-) fibroblasts tend to undergo adipogenic differentiation, contributing to orbital fat expansion, while CD90 (+) fibroblasts are more prone to fibrosis (13, 14). This dual differentiation potential explains the heterogeneity in clinical presentations, ranging from proptosis-dominant to fibrotic restrictive forms of GO.

### 2.3 Cytokine network and inflammatory cascade

The GO orbit exhibits infiltration by T cells (especially Th1 in the early stage and Th2 in later phases), B cells, and macrophages. These immune cells release a host of cytokines, including IL-6, IL-17, IFN-γ, and TNF-α, which further amplify local inflammation and fibroblast activation (15). TGF-β plays a pivotal role in sustaining chronic inflammation and fibrosis, and has emerged as an actively pursued therapeutic target (16). The activation of the JAK-STAT signaling pathway downstream of cytokine receptors further drives the pathological changes in the orbital tissues (17).

### 2.4 Role of B cells and autoantibodies

B cells contribute to the pathogenesis of GO through both antibody-dependent and independent mechanisms (18). In addition to producing TSIs and anti-IGF-1R antibodies, B cells act as antigen-presenting cells and secrete cytokines that shape T cell responses. Clinical studies with anti-CD20 monoclonal antibodies such as rituximab have confirmed the pathogenic relevance of B cells in moderate-to-severe GO (19, 20).

### 2.5 Oxidative stress and hypoxia

Oxidative stress also plays a critical role in GO, particularly in enhancing orbital fibroblast responses. Hyperglycemia, hypoxia, and smoking all increase reactive oxygen species (ROS) production, which activates proinflammatory transcription factors such as NF-κB (21). These factors synergize with cytokine signaling to exacerbate tissue damage. Moreover, hypoxia-inducible factor 1-alpha (HIF-1α) has been implicated in the pathological angiogenesis and tissue remodeling observed in GO (22).

## 3 Traditional pharmacological therapies for moderate-to-severe active graves’ ophthalmopathy

Despite recent advances in targeted therapies, traditional pharmacologic treatments remain foundational in the management of active GO. These treatments primarily focus on immune suppression, inflammation control, and symptomatic relief during the active phase of the disease.

### 3.1 Glucocorticoids

#### 3.1.1 Mechanism of action

Glucocorticoids exert potent anti-inflammatory and immunosuppressive effects by inhibiting the transcription of proinflammatory cytokines such as IL-1, IL-6, TNF-α, and IFN-γ, as well as reducing lymphocyte proliferation and migration. In GO, they dampen the autoimmune activation of orbital fibroblasts and reduce edema and inflammatory cell infiltration (23).

#### 3.1.2 Administration routes and regimens

Intravenous methylprednisolone (IVMP) is currently considered the first-line therapy for moderate-to-severe active GO due to its superior efficacy and lower incidence of systemic side effects compared to oral prednisone (24, 25). The standard cumulative dose ranges from 4.5 to 7.5 g administered over 12 weeks, as supported by EUGOGO guidelines (26).

High-dose oral GCs are reserved for cases with contraindications to IV administration, although they are associated with a higher risk of adverse events such as osteoporosis, glucose intolerance, and weight gain (27).

#### 3.1.3 Efficacy and limitations

IVMP achieves a positive response in improving clinical activity scores (CAS), proptosis, and diplopia, particularly when initiated during the early active phase (28). However, some patients are refractory to GCs, and recurrence is not uncommon. Moreover, cumulative doses above 8 g are associated with hepatotoxicity and cardiovascular risks, limiting long-term use (29).

### 3.2 Radiotherapy

Orbital radiotherapy (ORT) delivers localized low-dose radiation (20 Gy over 10 sessions) to suppress lymphocytic infiltration and fibroblast activity (30, 31). ORT is often used as an adjunct to GCs, especially in cases with diplopia and extraocular muscle involvement. Although its anti-inflammatory effects are modest, combination therapy with IVMP can yield additive benefits. However, its use is limited by delayed onset of action and concerns over radiation-induced retinopathy or optic neuropathy, especially in diabetic patients (32).

### 3.3 Immunosuppressive agents

#### 3.3.1 Mycophenolate mofetil

MMF selectively inhibits inosine monophosphate dehydrogenase, thereby suppressing B and T cell proliferation. Recent meta-analysis suggests MMF, either alone or combined with GCs, is superior to GCs alone in improving CAS and reducing relapse (33, 34). MMF is well tolerated and represents a promising alternative or adjunct, particularly for steroid-resistant or relapsing cases.

#### 3.3.2 Cyclosporine and azathioprine

The use of some traditional nonspecific immunosuppressants such as mycophenolate, cyclosporine and azathioprine appears useful in combination with steroid therapy to achieve stable results in the long term (35). Therefore, these agents have historically been used as adjuncts to GCs in refractory cases, though their efficacy is limited and toxicity profiles (nephrotoxicity, hepatotoxicity, bone marrow suppression) restrict widespread use (36).

### 3.4 Rituximab

Rituximab, an anti-CD20 monoclonal antibody, depletes B cells and reduces autoantibody production (37). Clinical trials on rituximab have yielded mixed results. While some studies demonstrated significant improvement in CAS and disease stabilization (18, 38), limited studies focusing on the steroid-resistant subpopulation of GO patients have shown inconsistencies in the response to Rituximab treatment (39–41). The discrepancy may be due to variation in disease stage and baseline activity. Nevertheless, rituximab remains a potential option in steroid-refractory and relapsing GO, particularly with high B cell activity.

### 3.5 Limitations of traditional therapies

Traditional therapies, especially GCs and radiotherapy, are largely non-specific and associated with significant systemic toxicity. They also fail to address the underlying molecular drivers of GO such as TSHR and IGF-1R signaling. Moreover, a substantial subset of patients are resistant or intolerant to these interventions, highlighting the need for novel, targeted approaches.

## 4 Emerging targeted therapies for active graves’ ophthalmopathy

With increasing insights into the molecular mechanisms of GO, a range of targeted biologics and small molecule inhibitors have emerged as promising alternatives or adjuncts to traditional immunosuppressive therapy. These therapies aim to interfere with key pathogenic pathways such as the IGF-1R, interleukin signaling, and the Janus kinase/signal transducer and activator of transcription (JAK-STAT) pathway.

### 4.1 Teprotumumab: IGF-1R inhibition

#### 4.1.1 Mechanism of action

Teprotumumab is a fully human monoclonal antibody that inhibits IGF-1R, a receptor overexpressed in orbital fibroblasts of GO patients and functionally linked to the TSHR (42). Its blockade reduces fibroblast activation, hyaluronic acid production, and adipogenesis, key contributors to tissue expansion and inflammation in GO.

#### 4.1.2 Clinical efficacy

Teprotumumab has demonstrated unprecedented efficacy in clinical trials (43, 44). The Open-Label Clinical Extension Study (OPTIC-X) evaluated the safety and efficacy of teprotumumab in GO patients who were nonresponsive or experienced disease flare after prior treatment in the OPTIC trial. Among 37 prior placebo recipients, 89.2% became proptosis responders with results comparable to those in the original OPTIC trial, despite a longer median disease duration (12.9 vs. 6.3 months). Response durability was high, with maintained improvements in proptosis, CAS, and diplopia at 48 weeks. Of the five initial nonresponders to teprotumumab, two responded upon re-treatment. Additionally, 62.5% of prior responders who flared showed benefit from re-treatment. No new safety concerns emerged, though mild hearing impairments were observed, reinforcing the need for continued postmarketing surveillance. These findings support the benefit of teprotumumab even in later disease stages and in cases requiring re-treatment (45).

#### 4.1.3 Safety and limitations

The most common adverse events included muscle spasms, hyperglycemia, and hearing-related disorders (e.g., tinnitus, hearing loss), some of which may be irreversible. Teprotumumab’s high cost and limited availability currently restrict its global use, though its FDA approval marks a paradigm shift in GO treatment (46).

### 4.2 Tocilizumab: IL-6 receptor blockade

#### 4.2.1 Mechanism of action

Tocilizumab is a humanized monoclonal antibody targeting the IL-6 receptor. IL-6 plays a central role in GO pathogenesis by promoting Th17 cell differentiation, B cell activation, and cytokine release. Inhibiting IL-6 signaling mitigates orbital inflammation and fibrosis (47, 48).

#### 4.2.2 Clinical data

Recent systematic review analyzed 29 studies on tocilizumab for GO, mostly case reports and series, with only one randomized clinical trial (RCT) (49). Tocilizumab, primarily used in glucocorticoid-resistant or relapsing cases, showed effectiveness in reducing inflammation and proptosis, with a low relapse rate (8.2%) and no severe side effects reported (49). Although the only RCT found no statistically significant improvement at six months, a recent meta-analysis suggested that tocilizumab may be the most effective option for reducing proptosis (50). Further randomized trials comparing tocilizumab with other treatments are needed.

#### 4.2.3 Adverse effects

Reported side effects include elevated liver enzymes, gastrointestinal discomfort, and risk of infections (51). Regular monitoring is essential, especially in long-term use. Its off-label use for GO, lack of large-scale randomized trials, and high cost limits its broader adoption.

### 4.3 Janus Kinase inhibitors

Recent study used a mouse model to investigate the role of transmembrane protein 2 (TMEM2) in GO. TMEM2 expression was significantly reduced in GO orbital tissues. Functional assays showed that TMEM2 suppresses inflammation, oxidative stress, and adipogenesis in orbital fibroblasts, while activating the JAK/STAT pathway. *In vivo*, TMEM2 overexpression alleviated inflammation, adipose tissue expansion, and fibrosis (17). These findings suggest TMEM2 as a key regulator in GO pathogenesis and a potential therapeutic target.

### 4.4 Other investigational agents

#### 4.4.1 TNF-α inhibitors

Drugs such as etanercept and infliximab, targeting TNF-α, have been trialed in GO with limited and inconsistent results. Though conceptually attractive, their impact on orbital inflammation appears less robust, and risk of infections remains a concern (52).

#### 4.4.2 IL-1 and IL-17 inhibitors

Agents targeting IL-1β (anakinra) and IL-17A (secukinumab) are under investigation due to their roles in Th1/Th17-mediated autoimmunity (53).

## 5 Comparison of traditional and emerging treatments: efficacy, safety, and clinical implications

As the therapeutic landscape for active Graves’ ophthalmopathy evolves, clinicians are increasingly faced with choices between traditional immunosuppressive therapies and emerging targeted agents. A comprehensive comparison of their efficacy, safety profiles, indications, and limitations is essential for personalized and evidence-based treatment planning.

### 5.1 Efficacy

#### 5.1.1 Improvement in clinical activity score

Previous studies have reported that approximately 58–83% of patients receiving IVGC, compared to 51% among those treated with oral glucocorticoids (OGC) (54–56). Of which, in a RCT involving 70 patients with active moderate-to-severe GO, median CAS decreased from 5 to 2 in the IVGC group, while in the OGC group, it declined from 5 to 3 (54). Notably, 77% (27/35) of IVGC-treated patients showed a 3-point CAS reduction, compared to 51% (18/35) in the OGC group. Another RCT with 81 participants receiving IVGC reported reductions in mean CAS from 3.66 at baseline to 1.65 and 1.68 (in right and left eyes, respectively) after 36 weeks of treatment (57). A RCT involving 16 GO patients treated with IVGC reported that 75% experienced a ≥2-point reduction in CAS at 24 weeks, with 69% achieving CAS inactivation (<3) (56). Another RCT comparing IVGC plus atorvastatin versus IVGC alone showed that only 28% of the 39 patients receiving IVGC monotherapy achieved improvement in a composite endpoint (58). Additionally, in a larger RCT of 159 patients with active moderate-to-severe TED, three different cumulative IVGC doses (2.25 g, 4.98 g, and 7.47 g) were evaluated. At 12 weeks, 81–83% of those receiving the medium and high doses had CAS improvements of >2 points, compared to 58% in the low-dose group. Disease inactivation (CAS ≤2) was observed in 45–65% of patients, depending on the dose (55).

#### 5.1.2 Proptosis reduction

One of the most significant limitations of traditional treatments is their limited effect on proptosis. Teprotumumab uniquely demonstrates clinically meaningful and statistically significant proptosis reduction (-3.0 mm for phase 2 study; -3.32 mm for phase 3 study), approaching the effects of orbital decompression surgery (59). Neither IVGCs nor cyclosporine can achieve such anatomical improvements.

#### 5.1.3 Diplopia and visual Function

Diplopia often persists despite IVGC therapy. Tocilizumab and teprotumumab have shown improvements in extraocular muscle function and diplopia scores (60), suggesting that early intervention with targeted therapies may reduce the need for rehabilitative surgery.

### 5.2 Safety profile

#### 5.2.1 Systemic glucocorticoids

Although effective, IVGCs are associated with considerable side effects, including hyperglycemia, weight gain, hypertension, osteoporosis, and hepatic dysfunction. These risks are particularly concerning in elderly patients and those with comorbidities. A previous retrospective cohort study compared the metabolic, immunological, and therapeutic effects of 4-week versus 12-week IVG therapy in 48 patients with active moderate-to-severe GO. The 12-week group showed significant increases in glucose and lipid levels, indicating a higher metabolic burden. Both groups had reduced bone metabolism markers and autoantibody levels, though thyroglobulin antibodies declined significantly only in the 4-week group. While both regimens improved clinical outcomes similarly, the 4-week group had greater ADC improvement and fewer metabolic side effects, supporting its use in patients with metabolic risk (61).

#### 5.2.2 Traditional immunosuppressants

Agents such as azathioprine and cyclosporine carry risks of nephrotoxicity, hepatotoxicity, and increased susceptibility to infections (62). Long-term use often necessitates careful monitoring and dose adjustments.

#### 5.2.3 Targeted therapies

Biologics, while generally better tolerated in the short term, have specific risks. Teprotumumab is associated with hearing abnormalities and glycemic disturbances; tocilizumab may cause elevated liver enzymes and neutropenia; JAK inhibitors have been linked to thrombosis and reactivation of latent infections. Thus, targeted therapies require individualized risk-benefit assessment and close monitoring.

### 5.3 Route of administration and treatment burden

Traditional treatments like IVGCs and immunosuppressants require either inpatient administration or frequent monitoring. Teprotumumab is administered via intravenous infusion every three weeks, which may be burdensome. Oral agents such as tofacitinib (a JAK inhibitor) offer greater convenience but remain investigational in GO.

### 5.4 Indications and patient selection

Therapies for active GO vary by severity and patient factors, with IV glucocorticoids as first-line and alternatives like teprotumumab, tocilizumab, JAK inhibitors, and radiotherapy used based on response and comorbidities (**Table 1**).

**Table 1:** Therapeutic options for active Graves’ orbitopathy and their clinical considerations.

Therapy	Best suited for	Contraindications / Considerations
IVGCs	First-line for moderate-to-severe active GO	Diabetes, hepatic dysfunction, psychiatric disorders
Teprotumumab	Active GO with proptosis and/or steroid failure	Cost, access, hearing issues, pregnancy
Tocilizumab	Steroid-refractory inflammation, IL-6-driven disease	Active infection, liver dysfunction
JAK inhibitors	Off-label use, rapid inflammation control	Risk of thromboembolism, immunosuppression
Orbital radiotherapy	Adjunctive for motility issues or CAS reduction	Radiation sensitivity, smoking history

### 5.5 Long-term outcomes and surgery avoidance

Emerging data suggest that early initiation of biologics, particularly teprotumumab, may reduce the need for rehabilitative surgery by reversing or halting the anatomical and functional deterioration of orbital tissues. This represents a major advancement, as conventional approaches often require staged decompression, muscle correction, and eyelid surgery.

### 5.6 Cost-effectiveness and accessibility

The financial burden of biologics remains a significant barrier to widespread adoption. Teprotumumab is cost-prohibitive in many countries and not yet available outside the U.S. Conversely, IVGCs and immunosuppressants are inexpensive and widely accessible but less effective for key GO outcomes such as proptosis (63).

### 5.7 The limitations of drug therapy for GO

Pharmacological therapy remains the cornerstone of treatment for active moderate-to-severe GO, with systemic corticosteroids being the most widely used first-line option due to their potent anti-inflammatory effects. However, their benefits are often transient, with high relapse rates and significant adverse effects limiting long-term use. Immunosuppressive agents such as mycophenolate mofetil and cyclosporine have demonstrated varying degrees of efficacy, while newer biologics like teprotumumab have shown superior outcomes in randomized trials. Nevertheless, drug resistance, individual response variability, high costs, and limited accessibility, especially in low-resource settings, highlight the need for continued therapeutic innovation. Furthermore, current treatments often target inflammation but have limited impact on late-stage fibrotic changes, emphasizing the importance of early diagnosis and timely intervention.

## 6 Future directions and challenges

Despite notable advances in pharmacological therapies for active GO, significant challenges remain. GO is a complex autoimmune disorder characterized by dysregulated TSHR and IGF-1R signaling, elevated proinflammatory cytokines, and heterogeneous orbital fibroblast subsets, all of which offer therapeutic targets. Although biologics such as the IGF-1R inhibitor teprotumumab have shown promising efficacy, issues related to high cost, limited global availability, and variability in patient response underscore the unmet clinical need for novel and accessible therapeutic options. Emerging biologic strategies, such as TSHR antagonists, anti-cytokine agents (e.g., IL-1β, TNF-α inhibitors), and biosimilars of existing drugs—are under investigation, alongside innovative cell-based therapies including mesenchymal stem cells (MSCs) and exosome-based approaches.

To address the heterogeneity of GO, future management should prioritize individualized, mechanism-based treatments that balance efficacy, safety, and cost. Precision medicine approaches, incorporating biomarker-guided stratification, pharmacogenomics, and phenotype-specific therapy (targeting immuno-, fibrotic-, or adipogenic-dominant patterns), may offer more tailored and effective care. At the same time, the development of biosimilars, pricing reform, and wider guideline implementation are urgently needed to address global disparities in access to advanced treatments. Furthermore, long-term outcome monitoring, strategies for relapse prevention, and the evaluation of optimal combination regimens remain key areas of investigation. Multidisciplinary collaboration and the integration of digital health tools—including AI-assisted imaging analysis and mobile health platforms, will be instrumental in advancing personalized, cost-effective, and sustainable GO care.
